# Retinoblastoma in Dandy-Walker Syndrome

**DOI:** 10.7759/cureus.89663

**Published:** 2025-08-08

**Authors:** Neiwete Lomi, Deepsekhar Das, Bhavna Chawla, Ananya Parampalli Ravindra

**Affiliations:** 1 Ophthalmology, All India Institute of Medical Sciences, New Delhi, New Delhi, IND

**Keywords:** dandy-walker malformation, dandy-walker syndrome, ocular malignancy, pediatric ocular malignancy, retinoblastoma

## Abstract

Dandy-Walker syndrome (DWS), also referred to as Dandy-Walker malformation, is a rare congenital developmental anomaly characterized by enlargement of the posterior fossa, dilatation of the fourth ventricle, and cerebellar hypoplasia with upward rotation. Retinoblastoma is the most common primary intraocular malignancy in children and typically presents in the pediatric age group, with leukocoria and strabismus being common early signs. Although DWS and retinoblastoma are individually rare, their simultaneous occurrence is exceptionally uncommon. The etiologies of both disorders have been linked to abnormalities in chromosome 13q, raising a theoretical basis for their association. However, actual co-manifestation remains scarcely reported. This report discusses what is likely the first documented case from the Indian subcontinent of bilateral retinoblastoma occurring in a child diagnosed with DWS. The coexistence of these two rare entities within a single patient raises awareness about the need for heightened vigilance in evaluating children with congenital brain anomalies for potentially life-threatening ocular malignancies.

## Introduction

Dandy-Walker syndrome (DWS), also known as Dandy-Walker malformation, is a rare congenital developmental anomaly involving enlargement of the posterior fossa, cystic dilatation of the fourth ventricle, and hypoplasia or agenesis of the cerebellar vermis. First described in 1914 by Dandy and Blackfan [1], DWS affects normal central nervous system development and presents with a spectrum of neurological symptoms, including hypotonia, ataxia, seizures, and cognitive delays [2]. While some cases remain asymptomatic, others are diagnosed in the context of multiple congenital anomalies, including neural tube defects, cardiac malformations, and cleft palate [2,3].

Ophthalmic manifestations of DWS are well-documented, ranging from nystagmus and strabismus to coloboma, microphthalmia, and optic nerve atrophy [4-7]. These findings underscore the embryological and anatomical relationship between the central nervous system and the visual pathway.

Retinoblastoma is the most common primary intraocular malignancy in children and typically presents before the age of five [8]. It commonly arises from biallelic mutations in the RB1 gene on chromosome 13q14, resulting in uncontrolled proliferation of retinal cells [8]. Clinical presentation includes leukocoria, strabismus, and visual inattention. While the condition has been associated with central nervous system anomalies such as pinealoblastoma and corpus callosal defects [9], its association with DWS is exceptionally rare.

Rodjan et al. [10] reviewed brain MRI scans of 168 children with retinoblastoma over 20 years and identified only a single case associated with a Dandy-Walker variant, indicating an exceedingly low prevalence of this combination. Although both DWS and retinoblastoma may be linked to chromosome 13q abnormalities, their co-occurrence remains largely unexplored in the literature.

To the best of our knowledge, this is the first reported case of bilateral retinoblastoma in a child diagnosed with DWS from the Indian subcontinent. This unique presentation highlights the importance of a high index of suspicion and thorough systemic evaluation in children presenting with congenital anomalies and ocular abnormalities.

## Case presentation

An infant presented to the ophthalmology outpatient department with a history of a white reflex in the left eye observed by the parents. The child was full-term with normal perinatal history and development. General physical examination revealed a healthy infant with stable vital signs and no gross neurological deficits.

Ocular evaluation revealed that the child resisted occlusion of the right eye and followed objects only when the left eye was covered. A relative afferent pupillary defect (RAPD) was noted in the left eye. Distant direct ophthalmoscopy revealed a greyish-white reflex in the left eye (leukocoria).

**Figure 1:**
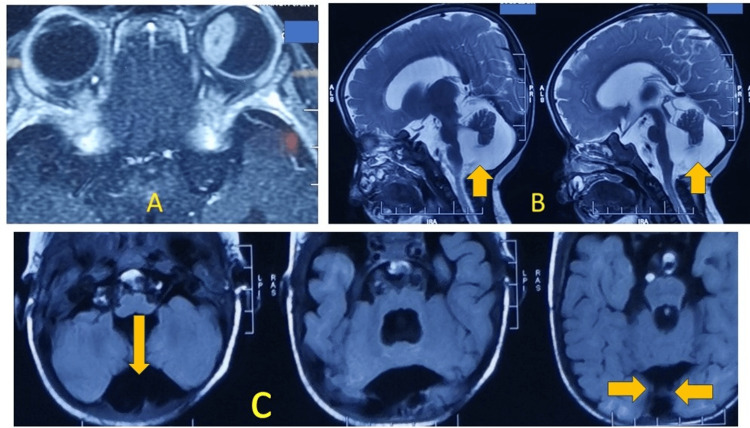
Contrast-enhanced magnetic resonance imaging (CEMRI) head and orbit A. CEMRI T1-weighted images of both eyes (axial sections) showing a large intraocular mass in the left eyeball. B. CEMRI T2-weighted images of the sagittal section showing enlargement of the posterior fossa (yellow arrows) with upward rotation of the cerebellum. C. CEMRI T1-weighted images of the axial section showing enlargement of the posterior fossa (yellow arrows) with hypoplasia of the cerebellar vermis.

Due to poor cooperation, examination under anesthesia (EUA) was scheduled. Ocular ultrasonography showed a small retinal lesion in the inferior quadrant of the right eye, while the left eye had a large intraocular lesion occupying nearly 40% of the globe, with evidence of calcification suggestive of retinoblastoma. Contrast-enhanced MRI of the brain and orbits confirmed enhancing intraocular lesions in both eyes, with retinal detachment in the left eye but no optic nerve involvement (Figure 1).

Notably, MRI also showed a cystic posterior fossa malformation and hypoplasia of the cerebellar vermis, consistent with DWS. A pediatric neurology consultation confirmed the diagnosis of DWS.

**Figure 2:**
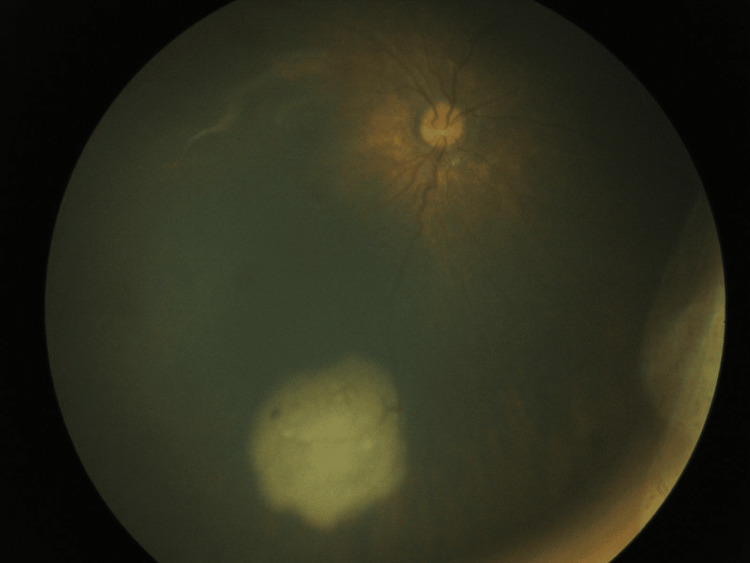
Fundus photograph of the right eye showing a Group B lesion in the inferior quadrant

On EUA, fundus examination revealed a 3 × 4 mm lesion in the inferior quadrant of the right eye and a large intraocular mass with vitreous seeding in the left eye. Based on clinical features and imaging, the diagnosis of bilateral retinoblastoma was made - classified as Group B in the right eye and Group E in the left, according to Murphree’s classification [9] (Figure 2).

**Figure 3:**
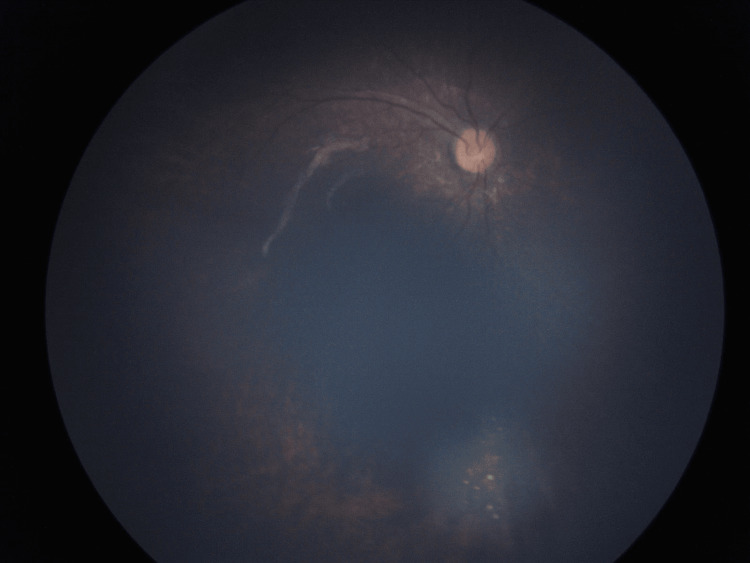
RetCam® image of the right eye showing a regressed lesion after six cycles of chemotherapy and transpupillary thermotherapy

Systemic chemotherapy using vincristine (1.5 mg/m^2^), etoposide (150 mg/m^2^), and carboplatin (560 mg/m^2^) was initiated. After three cycles, enucleation of the left eye was performed. The right eye was treated with transpupillary thermotherapy and three more chemotherapy cycles. At a nine-month follow-up, the child remains tumor-free with no recurrence or neurological deterioration (Figure 3).

## Discussion

The concurrent presentation of DWS and bilateral retinoblastoma is extraordinarily rare. Although both conditions have independently been linked to abnormalities on chromosome 13q, their combination is infrequently reported. This case adds to the limited body of evidence suggesting a possible association [9,10].

DWS results from disruptions during embryological development of the cerebellum and fourth ventricle, typically between the sixth and seventh gestational weeks [2]. It may exist as an isolated anomaly or alongside syndromic features involving the cardiovascular and craniofacial systems. The patient may have developmental delay, hypotonia, or hydrocephalus. Ophthalmic findings, such as colobomas, nystagmus, and optic nerve hypoplasia, are common in DWS, but intraocular malignancies are not [4-7].

Retinoblastoma arises from mutations in the tumor suppressor gene RB1, also located on chromosome 13q14 [8]. Bilateral retinoblastoma is often inherited and presents earlier than unilateral forms. Though the genetic basis for DWS is not fully elucidated, reports of its occurrence with chromosome 13 deletions suggest potential overlap in the genetic mechanisms underlying both conditions [10].

Rodjan et al. [10] reviewed MRIs of 168 children with retinoblastoma and found only one associated case of a Dandy-Walker variant. This low incidence emphasizes the novelty and clinical significance of the present report. To our knowledge, no previous cases of this combination have been documented in the Indian population.

From a clinical perspective, early detection is crucial in managing retinoblastoma, particularly in children with syndromic features where diagnostic overshadowing may delay recognition. In our case, prompt ocular imaging and MRI enabled early diagnosis and classification, guiding effective treatment. The use of chemotherapy and focal therapies preserved the right eye and vision, while enucleation controlled the advanced disease in the left eye.

This case underscores the importance of comprehensive systemic and neurological evaluation in children with ocular abnormalities. Pediatricians and ophthalmologists must maintain vigilance when encountering leukocoria in syndromic infants. Additionally, interdisciplinary collaboration plays a pivotal role in optimizing outcomes.

Further investigation, including genetic studies, may help clarify whether DWS and retinoblastoma are linked by shared mutations or arise independently. Documenting such rare associations enhances our understanding and may improve screening strategies for similar presentations.

Limitation

The parents of the patient did not consent to genetic testing, which otherwise would have provided a better understanding of the genes involved in this patient.

## Conclusions

This case represents a rare and possibly first reported instance of bilateral retinoblastoma in a child diagnosed with DWS from the Indian subcontinent. While both disorders have been associated with chromosomal aberrations involving 13q, their co-occurrence is exceptional, with only isolated global reports available. The case emphasizes the importance of early detection, interdisciplinary evaluation, and individualized treatment planning in managing complex pediatric conditions. A high index of suspicion is essential when infants present with ocular symptoms alongside neurological or developmental abnormalities. The use of imaging, timely chemotherapy, and surgical intervention in this patient led to favorable outcomes, including eye preservation and tumor-free survival over nine months of follow-up.

This report contributes to the limited existing literature on this unique association and underscores the need for further studies to explore potential genetic or developmental links. Broader awareness and documentation of such rare cases will aid clinicians in recognizing and managing similar complex scenarios more effectively.
